# Oral tuberculosis presenting as a destructive lower-lip ulcer: A case report

**DOI:** 10.1016/j.idcr.2026.e02771

**Published:** 2026-09-12

**Authors:** Mhd Anas Kattash, Karim Nahhas, Nour Waheeda Sabouni, Sulaima Kochaji, Nabil Kochaji

**Affiliations:** a Department of Oral Pathology, Faculty of Dentistry, Yarmouk Private University, Jabab, Daraa, Syrian Arab Republic; b Al-Qutayfah National Hospital, Ministry of Health, Al-Qutayfah, Damascus, Syrian Arab Republic; c Al-Kochaji Oral Maxillofacial Pathology Lab, Damascus, Syrian Arab Republic; d Faculty of Dentistry, Yarmouk Private University, Jabab, Daraa, Syrian Arab Republic; e Oral Histology and Pathology Department, Faculty of Dentistry, Damascus University, Damascus, Syrian Arab Republic

**Keywords:** Tuberculosis, Lip, Ulcer, Epithelial dysplasia, Granuloma, Mycobacterium tuberculosis complex

## Abstract

Oral and maxillofacial tuberculosis is uncommon, and labial involvement is unusual. This report describes lower-lip tuberculosis presenting as a destructive ulcer that clinically resembled malignancy and highlights the value of combined histopathological and microbiological assessment. A 24-year-old man presented with a three-month history of a progressively enlarging lower-lip ulcer causing functional interference and intermittent bleeding. Examination showed a solitary 18 × 11 mm ulcer with an irregular destructive surface, elevated margins, yellow–brown crusting and slough, haemorrhagic areas, and central necrosis. Incisional biopsy demonstrated mild epithelial dysplasia overlying necrotising granulomatous inflammation with Langhans-type multinucleated giant cells and caseous necrosis. No invasive carcinoma was identified in the examined tissue. A subsequent pretreatment biopsy submitted for mycobacterial culture yielded *Mycobacterium tuberculosis* complex (MTBC). Chest radiography showed no radiographic evidence of pulmonary tuberculosis, human immunodeficiency virus testing was negative, and no other tuberculous focus was identified on the available assessment. After two months of first-line treatment, the ulcer had decreased to approximately 5 × 3 mm. The case demonstrates that lower-lip tuberculosis can closely mimic malignancy. Necrotising granulomatous inflammation strongly supported tuberculosis but was not pathogen-specific, whereas microbiological confirmation established the aetiology. Concurrent mild epithelial dysplasia created an additional interpretative challenge because inflammatory and regenerative epithelial changes may overlap morphologically with dysplasia. Tuberculosis should remain in the differential diagnosis of persistent destructive lip ulcers. Representative tissue sampling, targeted microbiological investigation, and systemic assessment are essential for accurate diagnosis, appropriate classification, and avoidance of diagnostic closure.

## Introduction

Tuberculosis remains a major global infectious disease and, although it predominantly affects the lungs, virtually any anatomical site may be involved. In 2024, 8.3 million people with a new or recurrent episode of tuberculosis were diagnosed and notified worldwide, of whom 16% had extrapulmonary disease, underscoring the substantial global burden of extrapulmonary tuberculosis [1].

Oral tuberculosis is an uncommon manifestation of Mycobacterium tuberculosis infection that may occur as an isolated primary lesion or as secondary involvement associated with disease elsewhere. Its true frequency is difficult to determine because the published literature is dominated by case reports and small case series [[2], [3], [4]]. Ulceration is the most frequently reported clinical presentation, and although virtually any oral site may be affected, the tongue is the most commonly involved site, whereas labial involvement is infrequently reported [5,6]. Primary oral tuberculosis is also less commonly reported than secondary disease, making isolated primary tuberculosis of the lip an unusual presentation [[2], [3], [4]].

Beyond its rarity, oral tuberculosis presents an important diagnostic challenge because its clinical appearance is non-specific. A chronic destructive labial ulcer may resemble squamous cell carcinoma, traumatic ulceration, or another infectious or granulomatous disorder [2,4,7]. Histopathological findings such as necrotising granulomatous inflammation, Langhans-type giant cells and caseous necrosis may strongly suggest tuberculosis but are not pathogen-specific; microbiological investigation is therefore important for aetiological confirmation [8,9]. Systemic assessment is likewise essential because classification as primary or secondary oral tuberculosis depends on whether another focus of active disease is identified [2,4,7].

We report a case of isolated primary tuberculosis of the lower lip in a young adult, highlighting its clinical resemblance to malignancy and the unusual coexistence of microbiologically confirmed tuberculosis with mild epithelial dysplasia. This case report was prepared in accordance with the CARE (CAse REport) guidelines [10].

## Case presentation

A 24-year-old man presented with a three-month history of a progressively enlarging ulcer centred on the lower lip. The lesion interfered with lip function and bled intermittently on stimulation. He had no relevant medical history, reported no known tuberculosis contact, and denied smoking, medication use or preceding local trauma. No specific exposure associated with direct mucosal inoculation was identified in the available history. He also denied fever, weight loss, night sweats and chronic cough and had no known immunosuppressive condition.

Clinical examination disclosed a solitary ulcerative lesion involving the central lower lip, measuring approximately 18 × 11 mm. It had an irregular destructive surface with elevated margins, extensive yellow–brown crusting and slough, haemorrhagic areas, and a necrotic centre (Fig. 1). No additional intraoral lesions or palpable cervical lymphadenopathy were identified. The clinical differential diagnosis included squamous cell carcinoma, traumatic ulceration, and an infectious aetiology.

**Fig. 1:**
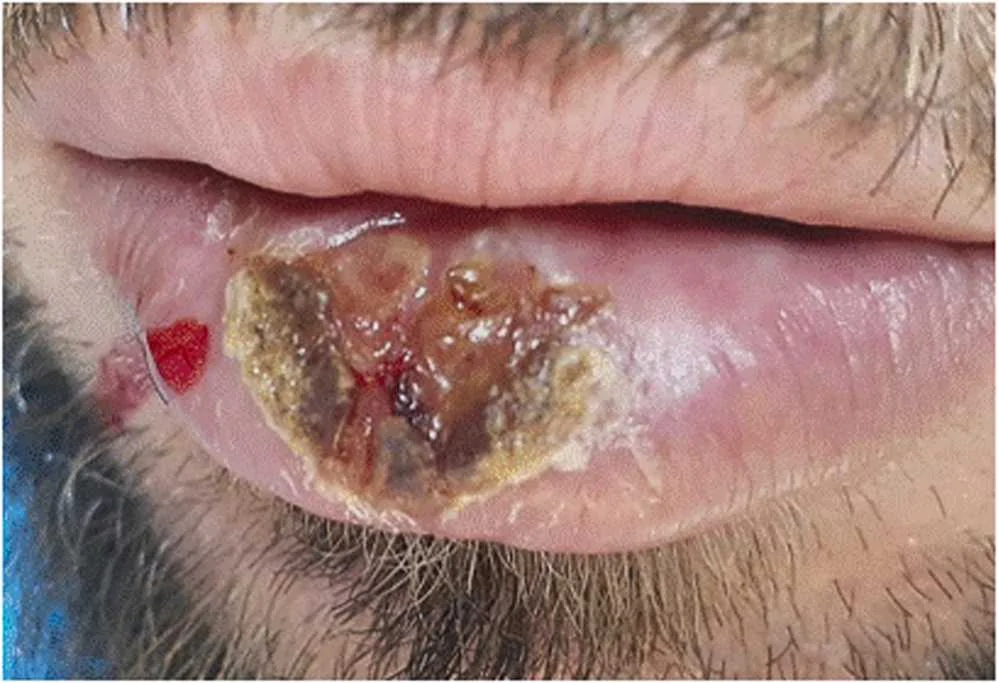
Clinical appearance of the lower-lip lesion. Frontal clinical view showing a solitary ulcer centred on the lower lip, with an irregular destructive surface, elevated margins, extensive yellow–brown crusting and slough, haemorrhagic areas, and central necrosis.

An incisional biopsy was performed the day after initial presentation and included the centre and margins of the lesion. Gross examination showed firm soft tissue measuring 10 × 11 × 5 mm, without grossly identifiable nodules or other suspicious features (Fig. 2).

**Fig. 2:**
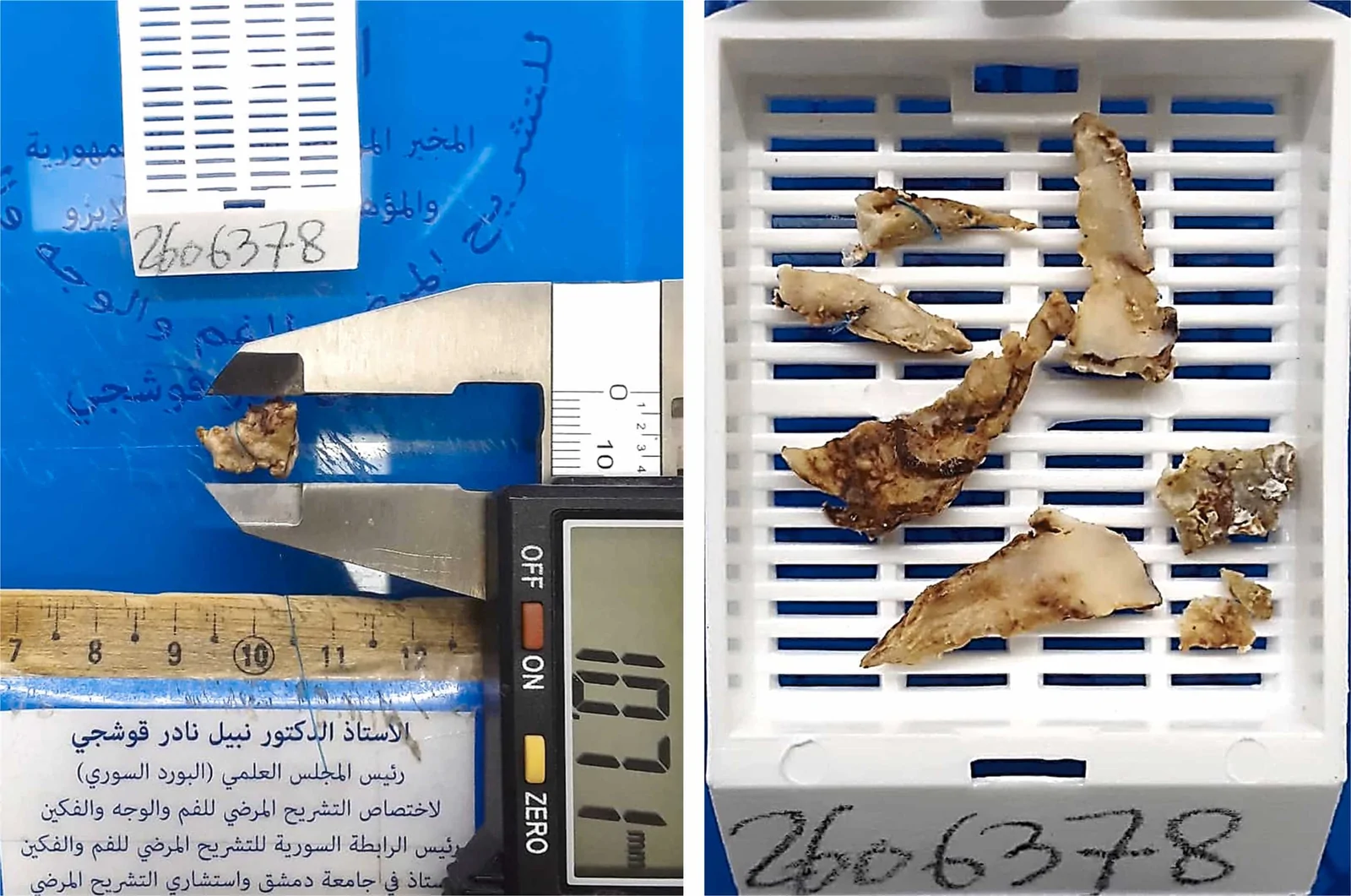
Gross appearance of the lower-lip incisional biopsy specimen. (A) Representative biopsy fragment measured with a digital calliper. (B) Multiple firm soft-tissue biopsy fragments arranged in a specimen cassette. No grossly identifiable nodules or other suspicious structures were observed.

Histopathological examination showed keratinised stratified squamous epithelium with mild epithelial dysplasia overlying inflamed connective tissue. The connective tissue contained necrotising granulomatous inflammation with numerous Langhans-type multinucleated giant cells and extensive areas of caseous necrosis (Fig. 3A–C). No invasive carcinoma was identified in the examined tissue. These findings were strongly suggestive of tuberculosis and prompted microbiological investigation. Ziehl–Neelsen staining of the initial paraffin-embedded biopsy was negative for acid-fast bacilli, while periodic acid–Schiff (PAS) and Grocott methenamine silver (GMS) stains showed no fungal organisms.

**Fig. 3:**
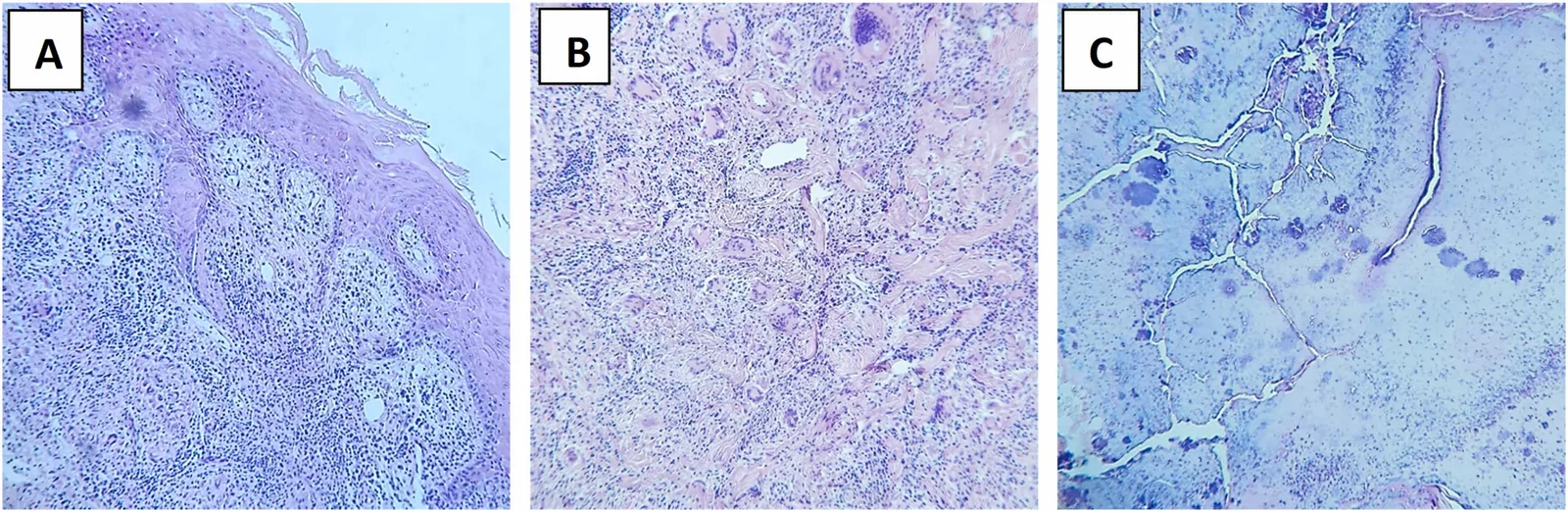
Histopathological features of the lower-lip biopsy. (A) Keratinised stratified squamous epithelium showing mild epithelial dysplasia (H&E stain, ×100). (B) Heavily inflamed connective tissue containing readily identifiable Langhans-type multinucleated giant cells (H&E stain, ×200). (C) Area of caseous necrosis within the inflamed connective tissue (H&E stain, ×200).

Following histopathological assessment, a second biopsy was obtained from the residual active lower-lip lesion before initiation of antituberculous therapy and submitted unfixed for mycobacterial culture at an external laboratory. According to the laboratory record, the tissue specimen was processed using the laboratory's standard NALC–NaOH decontamination procedure and cultured on Löwenstein–Jensen medium. Mycobacterial growth was detected after approximately four weeks. The cultured colonies were acid-fast on Ziehl–Neelsen staining and were identified as Mycobacterium tuberculosis complex (MTBC) using an MPT64 antigen immunochromatographic assay. No species-level identification within the MTBC was performed. Molecular testing of the cultured isolate for rifampicin resistance was subsequently performed using the Xpert MTB/RIF assay, and rifampicin resistance was not detected. No additional nucleic acid amplification tests were performed. Phenotypic drug-susceptibility testing and molecular testing for resistance to other antituberculous drugs were not performed. No dedicated fungal culture was performed.

Chest radiography showed no radiographic evidence of pulmonary tuberculosis, clinical assessment identified no other extrapulmonary tuberculous focus, and HIV testing was negative. The patient subsequently received standard first-line treatment with isoniazid, rifampicin, pyrazinamide, and ethambutol during the two-month intensive phase, with a planned four-month continuation phase of isoniazid and rifampicin. Treating-centre records confirmed completion of the intensive phase. At the two-month follow-up visit, the lower-lip ulcer measured approximately 5 × 3 mm compared with 18 × 11 mm at presentation, with resolution of bleeding and marked reduction in crusting, representing a substantial clinical response to treatment. No repeat microbiological sampling of the lip lesion was documented after initiation of treatment. The patient did not return to the treating centre after the two-month visit; it could therefore not be established whether the planned continuation phase was commenced or completed.

## Discussion

Oral tuberculosis is an uncommon manifestation of *Mycobacterium tuberculosis* infection and may closely mimic malignancy and other chronic ulcerative disorders [2,4,7]. In a systematic review of 301 cases of oral and maxillofacial tuberculosis, ulceration was the predominant presentation and the tongue the most frequently affected site [3]. Although labial tuberculosis has been described previously, the significance of the present case lies in the convergence of a destructive carcinoma-like lower-lip ulcer, necrotising granulomatous inflammation, microbiological confirmation of MTBC, and concurrent mild epithelial dysplasia. Among the published reviews and lip/labial tuberculosis reports considered [[2], [3], [4], [5], [6]], we did not identify a report describing this same combination of findings. The principal learning point is therefore not the diagnosis of tuberculosis itself, but the need to avoid diagnostic closure when microbiologically confirmed infection coexists with epithelial dysplasia in a lesion clinically suspicious for carcinoma.

The progressively enlarging ulcer, with elevated margins, bleeding, crusting, and necrosis, appropriately raised concern for squamous cell carcinoma despite the patient’s young age. Tuberculous oral ulcers are morphologically variable and may closely resemble carcinoma and other persistent ulcerative lesions. Clinical appearance therefore justified biopsy but could not establish the aetiology; definitive assessment of persistent or otherwise suspicious oral lesions requires appropriate tissue sampling and clinicopathological correlation [7,11]. As with other uncommon oral lesions, unusual clinical presentations may be difficult to classify on clinical grounds alone, reinforcing the importance of histopathological assessment in establishing the diagnosis [12]. In the present case, histopathology narrowed the differential diagnosis: necrotising granulomatous inflammation with Langhans-type giant cells and caseous necrosis strongly supports tuberculosis, but these findings are not pathogen-specific and may occur in other mycobacterial or deep fungal infections [[7], [8], [9]]. Granulomatous inflammation was reported in only about 63% of oral tuberculosis cases in the systematic review by Farias Gabriel et al. [3]. Histopathology should therefore guide targeted microbiological investigation rather than be regarded as pathogen-specific evidence [9].

In the present case, Ziehl–Neelsen staining of the initial paraffin-embedded biopsy was negative for acid-fast bacilli, while PAS and GMS stains showed no fungal organisms. Culture of a second pretreatment lesion-derived biopsy specimen yielded Mycobacterium tuberculosis complex (MTBC) on Löwenstein–Jensen medium, with acid-fast colonies identified using an MPT64 antigen immunochromatographic assay, thereby establishing the tuberculous aetiology. Histopathology and microbiology were complementary: histology characterised the tissue response and showed no invasive carcinoma in the examined tissue, whereas culture established the infectious aetiology. Molecular testing of the cultured isolate using Xpert MTB/RIF did not detect rifampicin resistance; phenotypic drug-susceptibility testing and molecular testing for resistance to other antituberculous drugs were not performed. This distinction is particularly important in unusual extrapulmonary lesions, where a characteristic tissue reaction alone does not establish the pathogen [9].

Classification as isolated primary oral tuberculosis is supported by the absence of respiratory or constitutional symptoms, negative HIV testing, and the absence of another identified tuberculous focus on the available clinical assessment and chest radiography [2,13]. A normal chest radiograph, however, does not completely exclude occult pulmonary tuberculosis. In this setting, “primary” should therefore be understood as the absence of another demonstrable focus of active disease rather than proof of direct mucosal inoculation; occult pulmonary or extrapulmonary disease cannot be excluded with absolute certainty [2,4]. No specific route of acquisition was identified from the available exposure history, and systematic evaluation of household contacts was not documented.

The concurrent mild epithelial dysplasia adds an important interpretative challenge. Regenerative atypia in ulcerated, inflamed mucosa can overlap morphologically with dysplasia, and grading of oral epithelial dysplasia is subject to interobserver variability [14,15]. The biopsy therefore demonstrates coexistence, not causation: tuberculosis cannot be assumed to have produced the dysplasia, but the dysplasia should not be dismissed as reactive solely because infection was confirmed. Likewise, microbiological confirmation of tuberculosis does not exclude a coexisting neoplasm. Kakisi et al. reported carcinoma at the same lesion site in approximately 3% of reviewed oral tuberculosis cases, although this should not be interpreted as a population incidence [2]. No invasive carcinoma was identified in the present biopsy, but that conclusion applies only to the examined tissue.

Representative sampling remains relevant even though the incisional biopsy included both the centre and margins of the lesion. Previous reports of oral and labial tuberculosis have shown that initial biopsies may miss the diagnostic granulomatous component, with the diagnosis becoming evident only after repeat or more extensive sampling [7,13]. The reverse concern also applies; demonstration of tuberculosis in a sampled area does not completely exclude a separate neoplastic component elsewhere in a heterogeneous lesion. Persistent ulceration, induration, or suspicious epithelial change after treatment should therefore prompt reassessment and, where indicated, repeat biopsy.

The substantial clinical response to antituberculous therapy provided additional clinical correlation. After completion of the two-month intensive phase, the ulcer had decreased from 18 × 11 mm to approximately 5 × 3 mm, with resolution of bleeding and marked reduction in crusting. The documented early response therefore complemented the histopathological and microbiological findings. Post-treatment microbiological reassessment of the lesion was not available.

The principal limitation of this report is the absence of longer-term follow-up, which prevented confirmation of completion of the planned continuation phase, complete lesion resolution, and reassessment of the epithelial dysplasia. Overall, this case highlights the risk of diagnostic closure when microbiologically confirmed tuberculosis coexists with dysplastic epithelial changes in a lesion clinically suspicious for carcinoma and underscores the importance of integrating clinical, histopathological, and microbiological findings.

## CRediT authorship contribution statement

**Mhd Anas Kattash:** Writing – original draft, Validation, Data curation. **Karim Nahhas:** Validation, Resources, Data curation. **Nour Waheeda Sabouni:** Visualization, Investigation. **Sulaima Kochaji:** Writing – review & editing, Validation. **Nabil Kochaji:** Validation, Supervision, Project administration.

## Consent to publish declaration

Written informed consent was obtained from the patient for publication of this case report and accompanying clinical photographs.

## Ethics approval

Ethical approval was not required for this single-patient case report in accordance with institutional policy.

## Funding

This research did not receive any specific grant from funding agencies in the public, commercial, or not-for-profit sectors.

## Declaration of Competing Interest

The authors declare that they have no known competing financial interests or personal relationships that could have appeared to influence the work reported in this paper.

## Data Availability

The data supporting the findings of this case report are available within the article. Additional data are available from the corresponding author upon reasonable request.
